# Early Surgical Decompression and Pulmonary Embolism Risk in Traumatic Cervical Spinal Cord Injury: A Propensity Score-Matched National Analysis

**DOI:** 10.1177/21925682261474150

**Published:** 2026-07-27

**Authors:** Samer G. Salman, Rohan A. Phadke, Nathan J. Lee

**Affiliations:** 1 School of Medicine, 3989 Baylor College of Medicine, Houston, TX, USA; 2 Midwest Orthopaedics at Rush, 12245 Rush University Medical Center, Chicago, IL, USA

**Keywords:** spinal cord injury, cervical spine, decompression, pulmonary embolism, venous thromboembolism, propensity score matching, national trauma data bank

## Abstract

**Study Design:**

Propensity score-matched retrospective cohort study.

**Objectives:**

To determine whether early cervical decompression is associated with in-hospital pulmonary embolism (PE) after traumatic cervical spinal cord injury (SCI).

**Methods:**

Adults with traumatic cervical SCI undergoing coded cervical decompression after direct admission were identified in the National Trauma Data Bank, 2019 to 2024. Early decompression was within 24 hours; delayed decompression was after 24 hours. Patients were matched 1:1 using 30 covariates. The primary outcome was detected in-hospital PE. Robustness analyses included sensitivity, subgroup, landmark, negative-control, competing-risk, clustered-error, bootstrap, and McNemar analyses.

**Results:**

Among 11,530 decompression-coded patients, matching yielded 4,480 balanced pairs. Early decompression was not associated with detected PE (OR 0.97, 95% CI 0.70 to 1.35; p = 0.868; absolute risk difference −0.04%, 95% CI −0.58% to 0.49%). Robustness analyses supported this null finding, including a null negative-control outcome (OR 1.10, 95% CI 0.75 to 1.62). No subgroup interaction was significant after multiplicity correction. In patients with Injury Severity Score ≥25, PE risk was similar (OR 0.88, 95% CI 0.56 to 1.39). Early decompression was associated with higher in-hospital mortality overall (OR 1.54, 95% CI 1.30 to 1.82), creating potential competing-risk bias.

**Conclusions:**

Early cervical decompression after traumatic cervical SCI was not associated with increased detected in-hospital PE. PE risk alone should not justify delaying otherwise indicated decompression, but differential mortality and residual confounding require cautious interpretation and prospective confirmation.

## Introduction

Traumatic cervical spinal cord injury (SCI) carries one of the highest risks of venous thromboembolism (VTE) among hospitalized patients. In untreated SCI populations, deep vein thrombosis (DVT) has been reported in 50% to 100% of patients, and pulmonary embolism (PE) remains a major cause of death.^1,2^ This risk reflects severe venous stasis from paralysis and sympathetic disruption, endothelial injury from trauma and surgery, and the hypercoagulable response to major injury.^3-6^ The American College of Surgeons Best Practices Guidelines for Spine Injury note that VTE risk is greatest early after injury, with most events occurring between 72 hours and 2 weeks, and recommend chemoprophylaxis as early as medically feasible, typically within 72 hours.^1^ Even with modern prophylaxis, PE still occurs in 0.4% to 1.8% of operative cervical SCI cohorts.^7,8^ Surgical decision-making in this population is further informed by injury-pattern severity, captured for subaxial fractures by the AO Spine Injury Score, which integrates morphology, neurology, and case-specific modifiers to guide operative urgency.^9^

Early decompression after acute SCI is supported by biologic rationale, prospective data, and clinical guidelines. In STASCIS, decompression within 24 hours was associated with higher odds of at least a two-grade improvement on the American Spinal Injury Association Impairment Scale at 6 months.^10^ A pooled analysis of four prospective datasets also found greater 1-year motor recovery with earlier surgery, with the largest decline in neurologic recovery occurring during the first 24 to 36 hours.^7^ Meta-analyses have further associated early decompression with improved neurologic outcomes, fewer complications, shorter hospitalization, and fewer pulmonary complications.^1,11,12^ Current AO Spine guidelines therefore conditionally recommend early surgery as a treatment option for adult acute SCI regardless of injury level, with operative approach and posterior subaxial instrumentation strategy informed by ongoing technical consensus in the cervical literature.^1,13,14^

Whether early decompression affects PE risk remains unclear. Earlier surgery could theoretically increase thrombotic risk through operative endothelial injury, perioperative immobility, and the physiologic stress of surgery in incompletely resuscitated trauma patients.^6^ Conversely, early decompression may reduce immobility, facilitate earlier mobilization, and shorten exposure to the acute stasis that drives VTE after SCI.^4^ Interpretation is further complicated because delayed surgery is often selected for patients with greater injury burden, physiologic instability, or comorbid conditions that also increase PE risk.^2^ To address this gap, we conducted a propensity score-matched retrospective cohort study using the National Trauma Data Bank to determine whether early cervical decompression, defined as surgery within 24 hours, is associated with in-hospital PE after traumatic cervical SCI and whether this association varies by injury severity.

## Methods

### Study Design and Data Source

We performed a retrospective cohort study using the National Trauma Data Bank (NTDB) Participant Use Files from 2019 through 2024.^15^ The NTDB, maintained by the American College of Surgeons, includes trauma registry data from more than 900 United States trauma centers. The Institutional Review Board (IRB) determined that use of the NTDB dataset does not constitute human subjects research and uses no identifiable protected health information. As a result, the project did not fall under the regulations for IRB review as defined in 45 CFR 46.

### Study Population

We included adults aged 18 years or older with traumatic cervical spinal cord injury (SCI) who underwent cervical spine surgery during the index hospitalization. Cervical SCI was defined using International Classification of Diseases, 10th Revision (ICD-10) codes S14.0xx and S14.1xx. Peripheral nerve injuries (S14.2-S14.9) were excluded because they do not involve the sympathetic disruption relevant to venous thromboembolism (VTE). Cervical procedures were identified using ICD-10 Procedure Coding System codes for fusion (0RG0-0RG4), reposition (0RS1-0RS6), and decompression (00NW, 009W, 009U).

**Figure 1.**
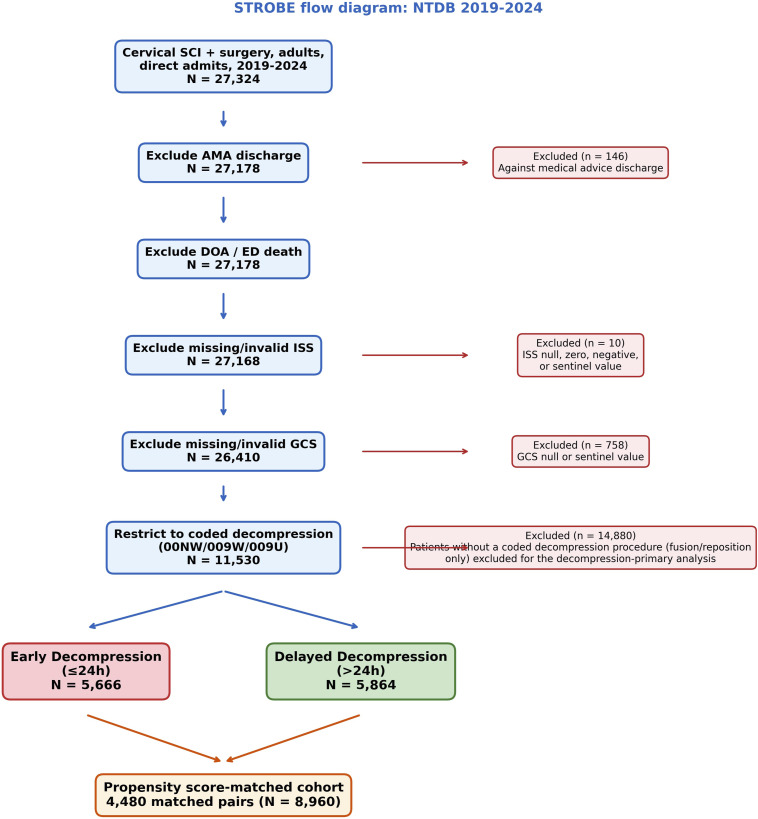
STROBE Flow Diagram for Cohort Selection and Propensity Score Matching. Sequential exclusion criteria were applied to 27,324 adult, directly admitted patients with traumatic cervical spinal cord injury who underwent cervical spine surgery in the National Trauma Data Bank from 2019 to 2024. After excluding against-medical-advice discharges, invalid or missing Injury Severity Score values, and invalid or missing Glasgow Coma Scale values, 26,410 operative cervical SCI patients remained. The primary analysis was then restricted to 11,530 patients with a coded cervical decompression procedure (ICD-10-PCS 00NW, 009W, or 009U), including 5,666 patients who underwent early decompression within 24 hours and 5,864 patients who underwent delayed decompression after 24 hours. Propensity score matching yielded 4,480 matched pairs, for a final matched analytic cohort of 8,960 patients

We included only direct admissions because transfer status can reset the recorded time from arrival to procedure. We excluded patients discharged against medical advice, patients dead on arrival or in the emergency department before surgery, and patients with missing or invalid Injury Severity Score (ISS) or Glasgow Coma Scale (GCS) score. Of 27,324 eligible adults, 146 were excluded for discharge against medical advice, 10 for missing or invalid ISS, and 758 for missing GCS, leaving 26,410 operative patients. Of these, 11,530 (43.7%) underwent a coded cervical decompression procedure (ICD-10-PCS 00NW, 009W, 009U) and comprised the primary analytic cohort; patients undergoing fusion or reposition without a coded decompression were excluded from the primary analysis (Figure 1). We also described nonoperative patients meeting the same criteria; 22,278 of 48,688 adult cervical SCI direct admissions (45.8%) did not undergo cervical spine surgery (Supplementary Table S1).

### Exposure

The exposure was time from hospital arrival to the first coded cervical decompression procedure (ICD-10-PCS 00NW, 009W, 009U). Early decompression was defined as decompression within 24 hours (≤24 hours), and delayed decompression as decompression after 24 hours (>24 hours), consistent with the STASCIS threshold. For patients with more than one decompression code, timing was assigned as the earliest recorded decompression. Per-procedure flags for decompression, fusion, and reposition were extracted from the procedure file so that the decompression-specific exposure could be defined; fusion and reposition codes were used only to characterize concomitant stabilization, not to define exposure timing.

### Outcomes

The primary outcome was in-hospital pulmonary embolism (PE), identified by TQIP hospital-event code 21 with a confirmed positive response. Secondary outcomes were deep vein thrombosis (DVT; code 14), composite VTE (PE or DVT), in-hospital mortality, acute respiratory distress syndrome (ARDS; code 5), sepsis, unplanned return to the operating room (code 30), unplanned ICU admission, cardiac arrest, ICU days, and ventilator days. Pneumonia was reported as ventilator-associated pneumonia (VAP; code 35).

The primary respiratory-utilization outcome was any mechanical ventilation, defined as ventilator days >0. Because missing ventilator days may indicate either no ventilation or no report, we also report conditional ventilator days among ventilated patients and sensitivity bounds assigning missing values to 0 days and to the cohort median (Supplementary Table S2). Catheter-associated urinary tract infection (CAUTI; code 33) was specified as a negative-control outcome because it is unlikely to be affected by decompression timing after adjustment. CAUTI was used instead of generic UTI because code 27 was empty.

### Covariates

Thirty covariates were selected a priori for the propensity score model because of clinical relevance to surgical timing and PE risk: demographics; emergency department vital signs and missingness indicators; ISS; upper (C1-C2) versus lower (C3-C7) cervical fracture level; traumatic brain injury; chest injury; congestive heart failure; chronic kidney disease; diabetes; hypertension; chronic obstructive pulmonary disease; cirrhosis; anticoagulant use; bleeding disorder; prehospital cardiac arrest; and functional dependence.

### Statistical Analysis

Continuous variables were summarized as median [interquartile range (IQR)], and categorical variables as frequency (percentage). Early and delayed groups were compared using standardized mean differences (SMDs), with SMD >0.1 indicating imbalance.

Propensity scores were estimated with multivariable logistic regression and used for 1:1 nearest-neighbor matching without replacement, with a caliper of 0.2 times the standard deviation of the logit propensity score. The propensity model C-statistic was reported. Postmatch balance required all covariates to have SMD <0.1.

The primary matched analysis estimated the association between early decompression and PE using logistic regression with Huber-White (HC1) robust standard errors for matched-pair correlation. Odds ratios (ORs), 95% confidence intervals (CIs), and two-sided p-values were reported. Absolute risk differences with 95% Wilson-Newcombe CIs were reported for PE overall and for the ISS ≥25 subgroup.

Dose response was assessed by modeling time to procedure with restricted cubic splines using knots at the 5th, 35th, 65th, and 95th percentiles. Because there was no evidence of nonlinearity, we also modeled time to procedure capped at 72 hours and reported the OR per 24-hour delay.

Five prespecified subgroup analyses tested effect modification by age (<65 vs ≥65 years), ISS (<25 vs ≥25), polytrauma pattern (isolated cervical SCI, SCI with traumatic brain injury, or SCI with chest injury), fracture level (C1-C2 vs C3-C7), and prehospital cardiac arrest. Interaction p-values came from multiplicative interaction terms. Bonferroni correction (α=0.01) and Benjamini-Hochberg false-discovery-rate correction (q=0.05) were applied to these tests.

Prespecified sensitivity analyses tested robustness by excluding withdrawal of life-sustaining treatment; restricting to 2023 to 2024; using 12-hour and 48-hour timing cutpoints; multivariable logistic regression without matching; stabilized inverse probability of treatment weighting trimmed at the 1st and 99th percentiles; excluding patients who died on ICU day 0; and using composite VTE. Because the primary analysis was restricted to coded cervical decompression, the coded-decompression sensitivity analysis was subsumed by the primary cohort.

Additional robustness analyses included cluster-robust standard errors by matched-pair ID, a 1,000-iteration pair-resampling bootstrap, and the McNemar exact test on discordant pairs. Landmark analyses at 48 and 72 hours restricted the PE model to patients with proxy survival to the landmark, defined as no death, ICU days at least the landmark day, or ventilator days at least the landmark day. Because NTDB lacks PE event dates, Fine-Gray analysis was not possible; we therefore reported stratified cumulative incidence of PE with death, death without PE, both, and neither by arm and in the ISS ≥25 subgroup.

The CAUTI negative-control outcome used the same matched logistic specification as PE. Missing ED vital signs were handled using missing indicators; we did not perform multiple imputation. E-values were calculated for the primary and key secondary outcomes. The plan was specified before cohort construction, but no formal pre-registration exists.

Analyses were performed in Python 3.x using pandas, statsmodels, and scikit-learn. A two-sided p-value <0.05 defined statistical significance for the primary outcome. All secondary outcomes, including in-hospital mortality, were prespecified but exploratory and were not adjusted for multiple comparisons; associations among them, including the mortality association, are hypothesis-generating and do not constitute causal evidence of surgical benefit or harm. Reporting followed STROBE guidelines.

## Results

### Study Population

The initial cohort included 27,324 adults with cervical SCI who underwent qualifying cervical spine surgery as direct admissions from 2019 to 2024. After sequential exclusions, 26,410 operative patients remained; 11,530 (43.7%) underwent a coded cervical decompression and formed the primary analytic cohort (Figure 1). Early decompression (≤24 hours) was performed in 5,666 patients (49.1%), and delayed decompression (>24 hours) in 5,864 patients (50.9%). Median time to decompression was 24.6 hours [IQR, 11.5-61.6]. The overall DVT rate was 519 (4.5%) (Table 1).

**Table 1.** Baseline Characteristics of the Study Cohort by Decompression Timing (Unmatched)

Variable	Total	Early	Delayed	*p- value*	SMD
N	11,530	5,666	5,864	—	​
Age, median [IQR]	61 [48-71]	59 [43-70]	63 [52-73]	<0.001	-0.303
ISS, median [IQR]	17 [16-26]	18 [16-26]	17 [16-24]	<0.001	0.217
GCS Total, median [IQR]	15 [14-15]	15 [14-15]	15 [15-15]	0.002	0.029
Time to Surgery (hours), median [IQR]	24.6 [11.5-61.6]	11.3 [6.4-17.5]	60.5 [38.0-107.6]	<0.001	-1.098
Female	2,647 (23.0%)	1,249 (22.0%)	1,398 (23.8%)	0.023	-0.043
Black Race	2,669 (23.1%)	1,272 (22.4%)	1,397 (23.8%)	0.084	-0.033
Hispanic Ethnicity	996 (8.6%)	514 (9.1%)	482 (8.2%)	0.111	0.030
Other Race/Ethnicity	992 (8.6%)	519 (9.2%)	473 (8.1%)	0.039	0.039
SBP < 90 mmHg	1,093 (9.5%)	635 (11.2%)	458 (7.8%)	<0.001	0.116
SBP Missing	105 (0.9%)	39 (0.7%)	66 (1.1%)	0.018	-0.046
HR ≥ 100 bpm	1,247 (10.8%)	520 (9.2%)	727 (12.4%)	<0.001	-0.104
HR Missing	99 (0.9%)	37 (0.7%)	62 (1.1%)	0.024	-0.044
GCS ≤ 8	847 (7.3%)	363 (6.4%)	484 (8.3%)	<0.001	-0.071
GCS 9-12	523 (4.5%)	295 (5.2%)	228 (3.9%)	<0.001	0.063
GCS Missing	0 (0.0%)	0 (0.0%)	0 (0.0%)	—	0.000
Temperature < 36 C	1,650 (14.3%)	915 (16.1%)	735 (12.5%)	<0.001	0.103
Temperature Missing	1,211 (10.5%)	631 (11.1%)	580 (9.9%)	0.031	0.041
Prehospital Cardiac Arrest	173 (1.5%)	77 (1.4%)	96 (1.6%)	0.250	-0.023
Withdrawal of Life-Sustaining Treatment	575 (5.0%)	343 (6.1%)	232 (4.0%)	<0.001	0.096
Upper Cervical (C1-C2)	1,536 (13.3%)	822 (14.5%)	714 (12.2%)	<0.001	0.069
Lower Cervical (C3-C7)	4,345 (37.7%)	2,548 (45.0%)	1,797 (30.6%)	<0.001	0.299
Traumatic Brain Injury	2,540 (22.0%)	1,173 (20.7%)	1,367 (23.3%)	<0.001	-0.063
Chest Injury	1,822 (15.8%)	926 (16.3%)	896 (15.3%)	0.124	0.029
CHF	415 (3.6%)	146 (2.6%)	269 (4.6%)	<0.001	-0.108
CKD	182 (1.6%)	66 (1.2%)	116 (2.0%)	<0.001	-0.065
Diabetes Mellitus	2,464 (21.4%)	1,052 (18.6%)	1,412 (24.1%)	<0.001	-0.135
Hypertension	4,995 (43.3%)	2,168 (38.3%)	2,827 (48.2%)	<0.001	-0.202
COPD	612 (5.3%)	228 (4.0%)	384 (6.5%)	<0.001	-0.113
Cirrhosis	115 (1.0%)	49 (0.9%)	66 (1.1%)	0.189	-0.026
Anticoagulant Use	1,126 (9.8%)	435 (7.7%)	691 (11.8%)	<0.001	-0.139
Bleeding Disorder	94 (0.8%)	43 (0.8%)	51 (0.9%)	0.577	-0.012
Functionally Dependent	911 (7.9%)	332 (5.9%)	579 (9.9%)	<0.001	-0.150
Pulmonary Embolism	197 (1.7%)	99 (1.7%)	98 (1.7%)	0.808	0.006
Deep Vein Thrombosis	519 (4.5%)	284 (5.0%)	235 (4.0%)	0.011	0.048
Venous Thromboembolism	653 (5.7%)	355 (6.3%)	298 (5.1%)	0.007	0.051
ARDS	161 (1.4%)	97 (1.7%)	64 (1.1%)	0.006	0.053
Pneumonia	466 (4.0%)	275 (4.9%)	191 (3.3%)	<0.001	0.081
Sepsis	186 (1.6%)	101 (1.8%)	85 (1.4%)	0.179	0.026
Unplanned Return to OR	24 (0.2%)	9 (0.2%)	15 (0.3%)	0.348	-0.021
Unplanned ICU Admission	813 (7.1%)	313 (5.5%)	500 (8.5%)	<0.001	-0.118
Cardiac Arrest	495 (4.3%)	299 (5.3%)	196 (3.3%)	<0.001	0.095
In-Hospital Mortality	757 (6.6%)	445 (7.9%)	312 (5.3%)	<0.001	0.102
ICU Days, median [IQR]	7 [4-13]	7 [4-13]	7 [4-13]	0.318	0.040
Ventilator Days, median [IQR]	8 [3-19]	8 [3-19]	9 [3-19]	0.052	-0.001

Data are presented as median [IQR] for continuous variables and n (%) for categorical variables. Early = surgery within 24 hours; Delayed = surgery after 24 hours. ARDS = acute respiratory distress syndrome; bpm = beats per minute; C = Celsius; C1-C2 = first to second cervical vertebrae; C3-C7 = third to seventh cervical vertebrae; CHF = congestive heart failure; CKD = chronic kidney disease; COPD = chronic obstructive pulmonary disease; GCS = Glasgow Coma Scale; HR = heart rate; ICU = intensive care unit; IQR = interquartile range; ISS = Injury Severity Score; mmHg = millimeters of mercury; N = number; OR = operating room; SBP = systolic blood pressure; SMD = standardized mean difference.

In the broader 2019 to 2024 adult cervical SCI direct-admit pool, 22,278 of 48,688 patients (45.8%) did not undergo cervical spine surgery. Compared with the operative cohort, nonoperative patients had higher in-hospital mortality (14.2% vs 6.3%) and withdrawal of life-sustaining treatment (9.6% vs 4.7%), but lower observed PE (0.8% vs 1.8%). These patients were excluded from the primary analysis by design and are summarized in Supplementary Table S1.

### Propensity Score Matching

**Figure 2.**
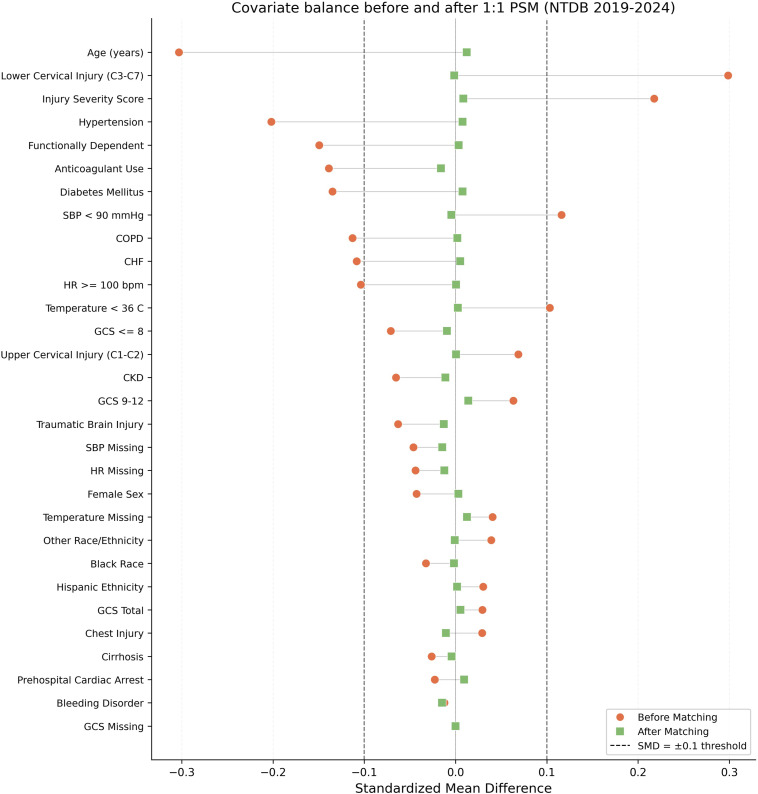
Covariate Balance Before and After 1:1 Propensity Score Matching. Love plot showing standardized mean differences for the 30 propensity-score covariates comparing early versus delayed cervical decompression. Orange circles show pre-match balance, green squares show post-match balance, and dashed vertical lines mark the ±0.10 balance threshold. All covariates were balanced after matching

One-to-one nearest-neighbor matching produced 4,480 matched pairs (N = 8,960). The unmatched propensity-score model had a C-statistic of 0.65. After matching, all 30 covariates were well balanced, with a maximum absolute SMD of 0.016 (all <0.1) (Table 2, Figure 2, Supplementary Figure S1).

**Table 2.** Covariate Balance before and after Propensity Score Matching

Variable	SMD Before	SMD After	Balanced
Age (years)	-0.3031	0.0123	Yes
Female Sex	-0.0427	0.0032	Yes
Black Race	-0.0326	-0.0016	Yes
Hispanic Ethnicity	0.0303	0.0016	Yes
Other Race/Ethnicity	0.0390	-0.0008	Yes
Injury Severity Score	0.2175	0.0085	Yes
GCS Total	0.0295	0.0054	Yes
GCS 9-12	0.0633	0.0140	Yes
GCS ≤ 8	-0.0709	-0.0095	Yes
GCS Missing	0.0000	0.0000	Yes
SBP < 90 mmHg	0.1160	-0.0047	Yes
SBP Missing	-0.0461	-0.0144	Yes
HR ≥ 100 bpm	-0.1039	0.0007	Yes
HR Missing	-0.0439	-0.0123	Yes
Temperature < 36 C	0.1033	0.0026	Yes
Temperature Missing	0.0406	0.0125	Yes
Prehospital Cardiac Arrest	-0.0229	0.0093	Yes
Upper Cervical Injury (C1-C2)	0.0686	0.0007	Yes
Lower Cervical Injury (C3-C7)	0.2987	-0.0014	Yes
Traumatic Brain Injury	-0.0630	-0.0129	Yes
Chest Injury	0.0292	-0.0105	Yes
CHF	-0.1083	0.0051	Yes
CKD	-0.0654	-0.0111	Yes
Diabetes Mellitus	-0.1349	0.0077	Yes
Hypertension	-0.2018	0.0077	Yes
COPD	-0.1130	0.0021	Yes
Cirrhosis	-0.0263	-0.0043	Yes
Anticoagulant Use	-0.1389	-0.0160	Yes
Bleeding Disorder	-0.0123	-0.0148	Yes
Functionally Dependent	-0.1495	0.0035	Yes
PS model C-statistic (original sample)	0.6511	0.6511	N/A

SMD = standardized mean difference. Balance threshold: |SMD| < 0.1. All 30 covariates achieved balance after matching (maximum |SMD| = 0.0160). bpm = beats per minute; C = Celsius; C1-C2 = first to second cervical vertebrae; C3-C7 = third to seventh cervical vertebrae; CHF = congestive heart failure; CKD = chronic kidney disease; COPD = chronic obstructive pulmonary disease; GCS = Glasgow Coma Scale; HR = heart rate; mmHg = millimeters of mercury; SBP = systolic blood pressure.

### Primary Outcome: Pulmonary Embolism

In the matched cohort, PE occurred in 73 early decompression patients (1.6%) and 75 delayed decompression patients (1.7%). Early decompression was not associated with PE overall (OR 0.97, 95% CI 0.70-1.35; p = 0.868). The absolute risk difference was -0.04% (95% CI -0.58% to +0.49%), a difference of negligible clinical magnitude (Table 3).

**Table 3.** Outcomes After Early versus Delayed Decompression in the Propensity-Matched Cohort

Outcome	Early (n=4,480)	Delayed (n=4,480)	OR	95% CI	*p-value*
**Thromboembolic outcomes**					
Pulmonary embolism	73 (1.6%)	75 (1.7%)	OR, 0.97	0.70 to 1.35	0.868
Deep vein thrombosis	224 (5.0%)	192 (4.3%)	OR, 1.18	0.96 to 1.43	0.108
Venous thromboembolism composite	273 (6.1%)	240 (5.4%)	OR, 1.15	0.96 to 1.37	0.134
**Major in-hospital outcomes**					
In-hospital mortality	360 (8.0%)	241 (5.4%)	OR, 1.54	1.30 to 1.82	<0.001
Acute respiratory distress syndrome	67 (1.5%)	56 (1.2%)	OR, 1.20	0.84 to 1.71	0.319
Ventilator-associated pneumonia	187 (4.2%)	158 (3.5%)	OR, 1.19	0.96 to 1.48	0.112
Catheter-associated urinary tract infection	55 (1.2%)	50 (1.1%)	OR, 1.10	0.75 to 1.62	0.624
Sepsis	75 (1.7%)	70 (1.6%)	OR, 1.07	0.77 to 1.49	0.676
Cardiac arrest	200 (4.5%)	164 (3.7%)	OR, 1.23	1.00 to 1.52	0.054
Unplanned return to the operating room	6 (0.1%)	11 (0.2%)	OR, 0.54	0.20 to 1.47	0.232
Unplanned intensive care unit admission	244 (5.4%)	365 (8.1%)	OR, 0.65	0.55 to 0.77	<0.001
**Resource-use outcomes**					
Intensive care unit days, median [IQR]	7 [4 to 12]	7 [5 to 13]	Median difference, 0.0	Mann-Whitney U	<0.001
Ventilator days, median [IQR]	7 [3 to 18]	9 [3 to 20]	Median difference, -2.0	Mann-Whitney U	<0.001
**Pulmonary embolism absolute risk-difference analyses**					
Pulmonary embolism, full matched cohort	73 (1.63%)	75 (1.67%)	RD, -0.045%	-0.579% to 0.489%	NA
Pulmonary embolism, ISS ≥25 subgroup	39 (2.93%)	38 (3.31%)	RD, -0.377%	-1.808% to 0.998%	NA

Values are presented as number (%) unless otherwise indicated. Binary outcomes were analyzed using logistic regression with heteroskedasticity-consistent type 1 robust standard errors. Continuous outcomes were compared using the Mann-Whitney U test and are reported as median [interquartile range]. Absolute risk differences are reported as early minus delayed, with Newcombe confidence intervals. CI = confidence interval; IQR = interquartile range; ISS = Injury Severity Score; NA = not applicable; OR = odds ratio; RD = risk difference.

**Figure 3.**
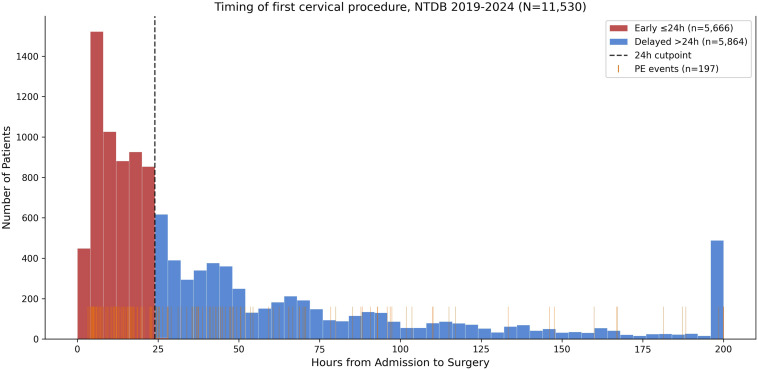
Timing of First Cervical Decompression Procedure. Histogram of admission-to-decompression time among 11,530 patients in the unmatched decompression-coded cohort, stratified by early (≤24 hours) and delayed (>24 hours) decompression. The dashed line marks the prespecified 24-hour exposure cutpoint. Orange rug marks indicate the surgery times of patients who developed in-hospital pulmonary embolism, not the timing of pulmonary embolism diagnosis

Results were unchanged across matched-pair inference checks. Cluster-robust standard errors, 1,000-iteration pair-resampling bootstrap, and McNemar exact testing all supported the primary estimate (cluster-robust OR 0.97, 95% CI 0.71-1.34, p = 0.867; bootstrap OR 0.97, 95% CI 0.70-1.34; McNemar p = 0.933) (Supplementary Table S3) (Figure 3).

Landmark analyses conditioning on proxy survival to 48 and 72 hours also matched the primary estimate (both OR 0.97, 95% CI 0.70-1.35), arguing against differential early attrition as the explanation for the null finding (Supplementary Table S4).

### Timing and Dose-Response

**Figure 4.**
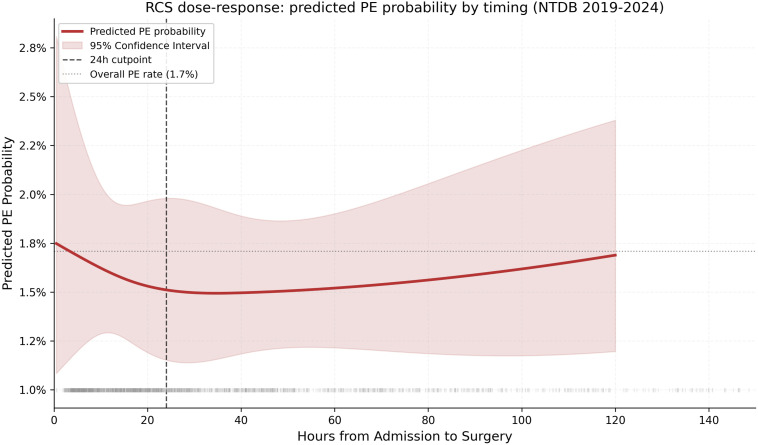
Continuous Association Between Surgical Timing and Predicted PE Risk. Restricted cubic spline model showing predicted in-hospital PE probability by hours from admission to cervical decompression. The shaded region shows the 95% CI, rug marks show observed procedure times, the dashed line marks the 24-hour cutpoint, and the dotted line shows the overall PE rate. Predicted PE risk remained low across timing, with greater uncertainty at the extremes

When time to procedure was modeled continuously and capped at 72 hours, each 24-hour delay was not associated with PE (OR 1.06, 95% CI 0.90-1.24; p = 0.492). Restricted cubic spline analysis showed a flat predicted-PE curve across the observed timing range, with no evidence of nonlinearity or threshold effect (Figure 4, Supplementary Table S2).

### Effect Modification

**Figure 5.**
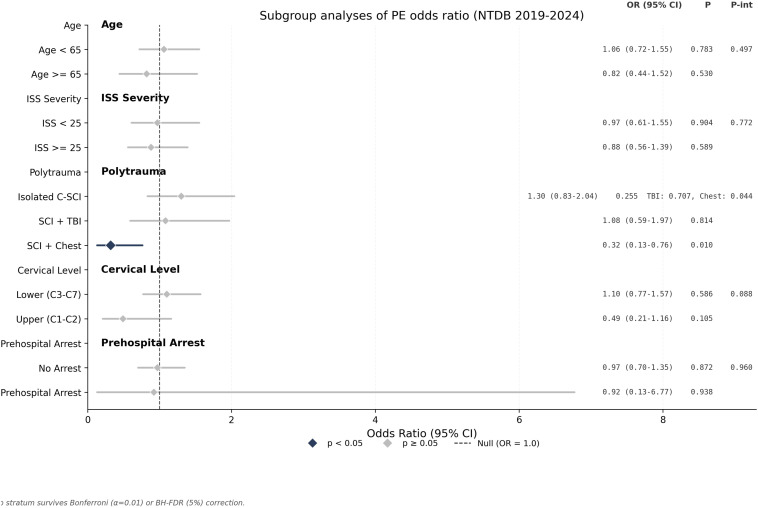
Forest Plot of Subgroup Analyses. Odds ratios with 95% CIs for the association between early decompression and PE across pre-specified subgroups. The dashed vertical line at OR = 1.0 indicates the null. Interaction p-values are shown on the right

None of the five prespecified subgroup interactions remained significant after Bonferroni correction (α = 0.01) or Benjamini-Hochberg false-discovery-rate correction (q = 0.05). The smallest interaction p-value was 0.044 for the chest-injury subgroup; because no interaction survived multiplicity correction, all within-stratum estimates should be considered hypothesis-generating (Table 4, Figure 5).

**Table 4.** Subgroup Analyses for the Association Between Early Decompression and Pulmonary Embolism

Subgroup	Stratum	N	Events	OR	95% CI	*p-value*	Interaction P
Age	Age < 65	5280	107	1.06	0.72–1.55	0.783	0.497
Age	Age ≥ 65	3680	41	0.82	0.44–1.52	0.53	0.497
ISS Severity	ISS < 25	6480	71	0.97	0.61–1.55	0.904	0.772
ISS Severity	ISS ≥ 25	2480	77	0.88	0.56–1.39	0.589	0.772
Polytrauma	Isolated C-SCI	6114	78	1.3	0.83–2.04	0.255	TBI: 0.707, Chest: 0.044
Polytrauma	SCI + TBI	1974	43	1.08	0.59–1.97	0.814	TBI: 0.707, Chest: 0.044
Polytrauma	SCI + Chest	872	27	0.32	0.13–0.76	0.01	TBI: 0.707, Chest: 0.044
Cervical Level	Lower (C3-C7)	7807	124	1.1	0.77–1.57	0.586	0.088
Cervical Level	Upper (C1-C2)	1153	24	0.49	0.21–1.16	0.105	0.088
Prehospital Arrest	No Arrest	8829	144	0.97	0.7–1.35	0.872	0.960
Prehospital Arrest	Prehospital Arrest	131	4	0.92	0.13–6.77	0.938	0.960

Interaction P values test for effect modification. Polytrauma interaction P reported separately for TBI and chest injury. C1-C2 = first to second cervical vertebrae; C3-C7 = third to seventh cervical vertebrae; CI = confidence interval; C-SCI = cervical spinal cord injury; ISS = Injury Severity Score; N = number; OR = odds ratio; SCI = spinal cord injury; TBI = traumatic brain injury.

In patients with ISS ≥25, early decompression was not significantly associated with PE (OR 0.88, 95% CI 0.56-1.39; p = 0.589), with an absolute risk difference of -0.38% (95% CI -1.81% to +1.00%). In patients with ISS <25, there was likewise no association (OR 0.97, 95% CI 0.61-1.55; p = 0.904). The lower PE odds previously suggested in this subgroup under a pooled cervical-surgery exposure were not observed when the exposure was restricted to coded decompression.

Within the ISS ≥25 stratum, in-hospital mortality was higher in the early decompression group (16.2% vs 11.9%; OR 1.42, 95% CI 1.13-1.79; p = 0.003), with an absolute risk difference of +4.23% (95% CI +1.48% to +6.94%) (Supplementary Table S4). This differential early mortality creates a competing-risk asymmetry, patients who die early have less opportunity to accrue a coded PE, and limits causal interpretation of any subgroup PE estimate.

In the chest-injury subgroup, lower PE odds were observed (OR 0.32, 95% CI 0.13-0.76; p = 0.010); however, the interaction p-value (0.044) did not survive multiplicity correction, so this remains hypothesis-generating and is not interpreted as a treatment effect.

### Secondary Outcomes

Any mechanical ventilation was more frequent after early decompression (39.5% vs 30.9%; OR 1.46, 95% CI 1.33-1.59; p <0.001). Among ventilated patients, ventilator duration was shorter in the early group (median 7 days [IQR, 3-18] vs 9 days [IQR, 3-20]; Mann-Whitney p <0.001). Sensitivity analyses for missing ventilator-day values did not change the any-ventilation finding (Supplementary Table S2).

Unplanned ICU admission was lower in the early decompression group (OR 0.65, 95% CI 0.55-0.77; p <0.001). DVT, composite VTE, ARDS, and sepsis did not differ significantly between groups (ARDS OR 1.20, 95% CI 0.84-1.71; p = 0.319). In-hospital mortality, a prespecified exploratory secondary endpoint, was higher after early decompression (OR 1.54, 95% CI 1.30-1.82; p <0.001; E-value 2.35 point estimate, 1.87 CI bound); because this is an exploratory, unadjusted association it does not by itself establish surgical harm, and it is addressed further in the Discussion.

As a prespecified negative-control outcome, CAUTI was not associated with early decompression (OR 1.10, 95% CI 0.75-1.62; p = 0.624) (Supplementary Table S5). A summary of all secondary outcomes is shown in Supplementary Figure S2.

### Sensitivity Analyses

**Figure 6.**
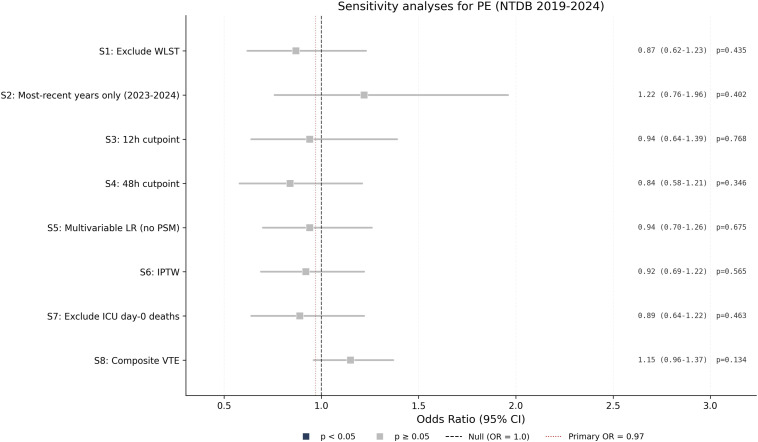
Sensitivity Analyses for Pulmonary Embolism. Forest plot showing odds ratios and 95% CIs for the eight sensitivity analyses comparing early versus delayed cervical decompression. The black dashed line denotes the null value (OR = 1.0), and the red dotted line denotes the primary matched-cohort estimate (OR = 0.97). Across sensitivity analyses, effect estimates remained close to the null and none reached statistical significance

All eight executed sensitivity analyses supported the null primary finding, with ORs ranging from 0.84 to 1.22 (Table 5, Figure 6). The E-value for the primary PE estimate was 1.20, with a CI-bound E-value of 2.01 (Supplementary Table S6).

**Table 5.** Sensitivity Analyses for the Association Between Early Decompression and Pulmonary Embolism

Analysis	Method	N	OR	95% CI	P value	Note
S1: Exclude WLST	PSM	10955	0.87	0.62–1.23	0.435	Removed 575 WLST patients
S2: Most-recent years only (2023-2024)	PSM	4859	1.22	0.76–1.96	0.402	Restricted to 2023-2024 (most-recent-years sensitivity under widened 2019-2024 window)
S3: 12h cutpoint	PSM	11530	0.94	0.64–1.39	0.768	Cutpoint ≤12h vs >12h
S4: 48h cutpoint	PSM	11530	0.84	0.58–1.21	0.346	Cutpoint ≤48h vs >48h
S5: Multivariable LR (no PSM)	Multivariable LR	11530	0.94	0.7–1.26	0.675	Adjusted for 29 covariates
S6: IPTW	IPTW	11530	0.92	0.69–1.22	0.565	Stabilized IPTW, trimmed 1st/99th (range: 0.62-2.07)
S7: Exclude ICU day-0 deaths	PSM	11517	0.89	0.64–1.22	0.463	Removed 13 patients who died on ICU day 0
S8: Composite VTE	PSM	11530	1.15	0.96–1.37	0.134	VTE (PE + DVT composite) as outcome

PSM = propensity score matching; LR = logistic regression; IPTW = inverse probability of treatment weighting. CI = confidence interval; h = hours; ICU = intensive care unit; N = number; OR = odds ratio; PE = pulmonary embolism; VTE = venous thromboembolism; WLST = withdrawal of life-sustaining treatment.

Excluding 575 patients (5.0%) who underwent withdrawal of life-sustaining treatment did not change the result (OR 0.87, 95% CI 0.62-1.23; p = 0.435). Restricting the analysis to the most recent years, 2023 to 2024, also supported the primary null finding (OR 1.22, 95% CI 0.76-1.96; p = 0.402).

## Discussion

In this propensity score-matched NTDB cohort of 11,530 patients undergoing coded cervical decompression (4,480 matched pairs), decompression within 24 hours was not associated with higher in-hospital PE risk. The matched estimate was null (OR 0.97, 95% CI 0.70-1.35), and this finding remained stable across cluster-robust standard errors, pair-resampling bootstrap, McNemar exact testing, landmark analyses at 48 and 72 hours, and eight estimable sensitivity analyses. Restricted cubic spline modeling also showed no dose-response relationship or threshold effect between surgical timing and PE. These findings provide reassurance that the established neurological rationale for early cervical decompression is not offset by a measurable increase in thromboembolic risk. ^7,11^

The null association is clinically plausible. Earlier surgery may reduce the duration of complete immobilization and permit earlier stabilization, mobilization, and coordinated postoperative care. ^4,7^ However, surgery also introduces transient prothrombotic exposures, including endothelial injury, operative positioning, and potential interruption or delay of chemoprophylaxis.^6^ The CLOTT study found that VTE prophylaxis initiation within 48 hours was associated with lower VTE risk in SCI patients, but the NTDB does not capture prophylaxis timing, agent, or adherence.^4^ These competing forces may explain why decompression timing itself was not independently associated with PE. The null CAUTI negative-control outcome is supportive of the internal consistency of the matched analysis. CAUTI is, however, an imperfect negative control in this ICU-level SCI population: catheter dwell time, ICU duration, and nursing care intensity may themselves be influenced by operative timing and postoperative trajectory. The null CAUTI result therefore provides supportive rather than definitive evidence against residual confounding by indication. ^7,13^

When the exposure was restricted to coded cervical decompression, we did not observe the lower PE odds in the ISS ≥25 subgroup that had been suggested under a pooled cervical-surgery exposure (OR 0.88, 95% CI 0.56-1.39; interaction p = 0.77), and no subgroup interaction survived multiplicity correction. Any within-subgroup PE estimate is therefore best regarded as hypothesis-generating. Mortality was higher in the early decompression arm within this subgroup (16.2% vs. 11.9%; OR 1.42), creating a competing-risk concern: patients who die early have less time to accrue a coded PE. This pattern is consistent with confounding by indication, in which physiologically unstable patients are more likely to undergo urgent surgery and also more likely to die. ^2,7^ Because the NTDB lacks PE event dates, formal time-to-event or Fine-Gray competing-risk modeling was not possible; we instead report stratified cumulative incidence of PE and death by arm (Supplementary Table S4). Subgroup PE findings should not change clinical practice without prospective validation using time-stamped PE events.

Secondary outcomes should be interpreted in the same clinical context. The higher rate of any mechanical ventilation in the early group likely reflects planned perioperative intubation and postoperative monitoring rather than increased respiratory morbidity, because ventilator duration among ventilated patients was shorter after early surgery. This pattern aligns with prior work suggesting that early stabilization may reduce pulmonary morbidity and ventilator days despite greater early ventilator use. ^4,12,16^ The lower rate of unplanned ICU admission after early surgery likely reflects planned ICU-level management around urgent operative care. By contrast, the higher overall in-hospital mortality in the early group is most consistent with residual confounding by indication rather than a harmful effect of early decompression. Mortality was a prespecified exploratory endpoint and was not adjusted for multiplicity; the association should be read as a marker of baseline illness severity that selected patients for urgent surgery, not as causal evidence that earlier decompression increases death. Important unmeasured variables, including ASIA grade, hemodynamic instability, vasopressor needs, goals-of-care decisions, and early withdrawal of life-sustaining treatment, could plausibly influence both timing and mortality. ^2,17,18^

This study has several strengths relevant to administrative trauma research. Matching used 30 covariates and achieved excellent measured balance, with a maximum post-match SMD of 0.016. ^19^ The analysis also tested the primary finding across multiple specifications, landmark cohorts, dose-response models, and a prespecified negative-control outcome. These features reduce the likelihood that the primary null finding is a product of model choice alone. However, the NTDB cannot capture several variables central to PE risk after cervical SCI, including chemoprophylaxis timing and agent, ASIA impairment grade, mean arterial pressure targets, neurological recovery, operative details, and goals-of-care decisions.^7^ The absence of granular SCI severity grading is a recognized limitation of registry-based SCI research, and proposed simplified classifications have been advanced specifically to enable real-time analytics and outcomes modeling that current registries cannot support. ^7,20^ The database also lacks PE event timing, preventing formal competing-risk analysis. Surgical timing was measured from hospital arrival rather than injury, which may misclassify patients with long prehospital intervals. Restricting the cohort to direct admissions reduced transfer-related timing artifacts but limits generalizability to transferred patients. ^18^ Finally, PE ascertainment depends on clinical detection and coding, which may underestimate true event rates and introduce surveillance bias.

For spine surgeons and trauma teams, the practical message is straightforward: PE risk should not be used as a reason to delay indicated early decompression in traumatic cervical SCI. Early surgery did not increase PE risk in the matched cohort, and no timing threshold was identified. At the same time, the overall PE rate of approximately 1.6% to 1.7% confirms that these patients remain at high thromboembolic risk regardless of operative timing, consistent with prior cervical spine series identifying extremity paralysis and central venous access as among the strongest preoperative VTE predictors. ^1,7,21^ Early decompression should therefore be paired with aggressive, protocolized VTE prevention once medically safe, as recommended in elective spine surgery consensus reviews, rather than viewed as a substitute for prophylaxis. ^22^ Future studies should link operative timing, prophylaxis timing, ASIA grade, mortality timing, and PE event timing to determine whether selected high-risk subgroups benefit from more intensive VTE prevention. These efforts align with international calls for sustained investment in evidence-based SCI care infrastructure. ^23^

## Conclusion

In this propensity score-matched cohort of patients undergoing cervical decompression for traumatic cervical SCI, decompression within 24 hours was not associated with an increase in detected in-hospital PE. This finding remained consistent across robustness analyses, landmark cohorts, dose-response modeling, and a negative-control outcome. No subgroup, including ISS ≥25, showed a PE interaction that survived multiplicity correction. The findings should be interpreted in the context of higher early mortality in the early-surgery group, which reflects confounding by indication and an unavoidable competing-risk asymmetry given the absence of PE event dates. These results support current practice favoring early cervical decompression when clinically indicated and do not support delaying surgery because of PE concern alone. Future studies with granular prophylaxis data, ASIA impairment grade, and time-stamped PE and mortality events are needed to clarify thromboembolic risk in this population.

## Supplemental Material

Supplemental material - Early Surgical Decompression and Pulmonary Embolism Risk in Traumatic Cervical Spinal Cord Injury: A Propensity Score-MMatched National Analysis Supplemental material for Early Surgical Decompression and Pulmonary Embolism Risk in Traumatic Cervical Spinal Cord Injury: A Propensity Score-MMatched National Analysis by Samer G. Salman, Rohan A. Phadke, and Nathan J. Lee in Global Spine Journal.
