# Supplementary material for: Early Surgical Decompression and Pulmonary Embolism Risk in Traumatic Cervical Spinal Cord Injury: A Propensity Score-Matched National Analysis
Source: Global Spine J. 2026 Jul 27:21925682261474150. Online ahead of print. doi: 10.1177/21925682261474150 (PMC13407683; doi:10.1177/21925682261474150)
Supplement: Supplemental material - Early Surgical Decompression and Pulmonary Embolism Risk in Traumatic Cervical Spinal Cord Injury: A Propensity Score-MMatched National Analysis [file sj-pdf-1-gsj-10.1177_21925682261474150.pdf]

Digital Supplementary Content

Early Cervical Decompression and Pulmonary Embolism Risk After Traumatic Cervical Spinal Cord Injury: A Propensity Score-Matched National Trauma Data Bank Analysis

Content:

Supplemental Table S1. Non-Operative versus Operative Adult Cervical SCI Direct Admits (NTDB 2019–2024)
Supplemental Table S2. Any-Ventilation Primary Outcome and Vent-Day Missingness Sensitivity Bounds
Supplemental Table S3. Cluster-Robust Standard Errors, Pair-Resampling Bootstrap, and McNemar Exact Test (Primary PE Outcome)
Supplemental Table S4. Competing-Risk Cross-Tabulation and Landmark (48h / 72h) Analyses
Supplemental Table S5. Negative-Control Outcome (Catheter-Associated UTI, NTDB Code 33)
Supplemental Table S6. E-Values for Key Outcomes (reference)
Supplemental Table S7. Prespecified but Unexecuted Analyses (Methodological Note)

Supplemental Figure S1. Propensity Score Distribution Overlap
Supplemental Figure S2. Forest Plot of Secondary Outcomes

Supplemental Table S1. Non-Operative versus Operative Adult Cervical SCI Direct Admits (NTDB 2019–2024)

metric	operative	nonoperative	Absolute difference	relative difference pct
N	26,410	22,278	+4,132	—
Pct of cervical SCI adult direct admits	54.2%	45.8%	—	—
Age (mean +/- SD)	56.3 +/- 17.6	55.1 +/- 19.7	+1.2	+2.2%
ISS (median [IQR])	17 [16-26]	17 [10-27]	+0.0	—
GCS (median [IQR])	15 [14-15]	15 [11-15]	+0.0	—
Female	6,185 (23.4%)	6,099 (27.4%)	-4.0 pp	—
PE	475 (1.8%)	179 (0.8%)	+1.0 pp	—
Mortality	1,671 (6.3%)	3,156 (14.2%)	-7.8 pp	—
Any ventilation (vent days > 0)	9,755 (36.9%)	6,281 (28.2%)	+8.7 pp	—
WLST	1,234 (4.7%)	2,129 (9.6%)	-4.9 pp	—
ICU days (median [IQR])	7 [4-13]	4 [2-8]	—	—

Non-operative = adults meeting identical cervical SCI, age, direct-admit, and exclusion criteria but without any cervical spine procedure. Provided to characterize selection into the operative primary cohort. Em dash (—) denotes not applicable. GCS = Glasgow Coma Scale; ICU = intensive care unit; IQR = interquartile range; ISS = Injury Severity Score; N = number; NTDB = National Trauma Data Bank; PE = pulmonary embolism; Pct = percentage; pp = percentage points; SCI = spinal cord injury; SD = standard deviation; vent days = ventilator days; WLST = withdrawal of life-sustaining treatment.

Supplemental Table S2. Any-Ventilation Primary Outcome and Vent-Day Missingness Sensitivity Bounds

analysis	arm early summary	arm delayed summary	estimate or or difference	ci or iqr	*p-value*
Any mechanical ventilation (vent days>0; NTDB: missing=not ventilated)	1,768/4,480 (39.5%)	1,386/4,480 (30.9%)	OR=1.46	(1.33-1.59)	<0.001
Conditional vent days (ventilated only, non-missing)	7 [3-18]	9 [3-20]	-2.0 (median diff)	(Mann-Whitney)	<0.001
Sensitivity 1: missing vent days=0 (cohort-wide median vent days)	0 [0-4]	0 [0-3]	+0.0 (median diff)	(Mann-Whitney)	<0.001
Sensitivity 2: missing vent days=cohort median (8.0) (median vent days)	8 [8-8]	8 [8-8]	+0.0 (median diff)	(Mann-Whitney)	<0.001

Missing vent-days bounded under missing = 0 (structural zero) and missing = cohort median; the any-ventilation odds-ratio estimate is unchanged under both assumptions. Dose-response: modeling time to procedure continuously (restricted cubic spline; Figure 4) showed a flat predicted-PE curve with no threshold effect, and the linear trend over 0–72 hours was not significant (OR 1.06 per 24 hours, 95% CI 0.90–1.24). CI = confidence interval; diff = difference; IQR = interquartile range; NTDB = National Trauma Data Bank; OR = odds ratio; vent days = ventilator days.

Supplemental Table S3. Robustness Analyses for the Primary Pulmonary Embolism Outcome

Robustness analysis	Effect estimate or test statistic	95% CI	*p-value*	Notes
Cluster-robust logistic regression by matched-pair identifier	OR 0.97	0.71 to 1.34	0.867	Logistic regression with standard errors clustered by matched-pair identifier.
Pair-resampling bootstrap of matched pairs	OR 0.97	0.70 to 1.34	0.856	Bootstrap distribution generated from 1,000 matched-pair resamples. The two-sided empirical *p-value* was calculated relative to the null value of OR = 1.
Exact McNemar test using pair-level discordance	Early decompression only: 70; delayed decompression only: 72	NA	0.933	Exact two-sided binomial test based on discordant pairs. Concordant pairs included 3 pairs with PE in both groups and 4,335 pairs with PE in neither group.

All three matched-cohort robustness analyses support the primary null inference for pulmonary embolism. The McNemar analysis was based on 4,480 matched pairs: 70 pairs in which PE occurred only in the early-decompression patient, 72 pairs in which PE occurred only in the delayed-decompression patient, 3 pairs in which PE occurred in both patients, and 4,335 pairs in which PE occurred in neither patient.

Abbreviations: CI, confidence interval; NA, not applicable; OR, odds ratio; PE, pulmonary embolism.

Supplemental Table S4. Competing-Risk Cross-Tabulation and Landmark (48h / 72h) Analyses

analysis	group	n	pe events	deaths	or estimate	ci low	ci high	abs risk diff pct	rd ci low	rd ci high	*p-value*	notes
Mortality in ISS>=25 subgroup	Overall	2480	—	352	1.42	1.13	1.79	+4.23	+1.48	+6.94	0.003	Early deaths 215/1331 vs Delayed 137/1149
Cumulative-incidence cross-tab	Matched overall: Early	4480	73	360	—	—	—	—	—	—	—	PE only=54; Death no PE=341; Both=19; Neither=4066
Cumulative-incidence cross-tab	Matched overall: Delayed	4480	75	241	—	—	—	—	—	—	—	PE only=65; Death no PE=231; Both=10; Neither=4174
Cumulative-incidence cross-tab	ISS>=25: Early	1331	39	215	—	—	—	—	—	—	—	PE only=28; Death no PE=204; Both=11; Neither=1088
Cumulative-incidence cross-tab	ISS>=25: Delayed	1149	38	137	—	—	—	—	—	—	—	PE only=32; Death no PE=131; Both=6; Neither=980
Landmark 48h PE (proxy)	Matched cohort	8949	148	—	0.97	0.70	1.35	—	—	—	0.872	proxy based=TRUE; survived to landmark proxied by (died==0 OR icu days>=2 OR vent days>=2)
Landmark 72h PE	Matched cohort	8939	148	—	0.97	0.70	1.35	—	—	—	0.874	proxy based=TRUE; survived to landmark proxied by (died==0 OR

(proxy)												icu days>=3 OR vent days>=3)
NOTE: Fine-Gray competing-risk regression	—	—	—	—	—	—	—	—	—	—	—	Not performed: NTDB 2019-2024 has no event-time data (no date/time of death or of PE diagnosis).

Fine-Gray subdistribution-hazard modeling was not performed because NTDB does not record event dates for PE. Landmark proxy uses (died = 0) OR (ICU days ≥ landmark day) OR (ventilator days ≥ landmark day). Em dash (—) denotes not applicable. abs = absolute; CI = confidence interval; h = hours; ICU = intensive care unit; ISS = Injury Severity Score; n = number; NTDB = National Trauma Data Bank; OR = odds ratio, except in the landmark proxy definition, where OR denotes logical “or”; PE = pulmonary embolism; pct = percentage; RD = risk difference.

Supplemental Table S5. Negative-Control Outcome (Catheter-Associated UTI, NTDB Code 33)

outcome label	n early	events early	rate early	n delayed	events delayed	rate delayed	or	ci low	ci high	*p-value*	interpretation
Catheter-Associated UTI (CAUTI, NTDB code 33)	4480	55	1.23%	4480	50	1.12%	1.10	0.75	1.62	0.626	Null OR consistent with no residual confounding by indication on CAUTI (biologically implausible to be affected by timing of cervical decompression).

Generic UTI code 27 was empty across the 2019–2024 PUF; CAUTI code 33 was used as the operational negative control. A null estimate supports no residual confounding on an outcome biologically unrelated to decompression timing. CAUTI = catheter-associated urinary tract infection; CI = confidence interval; n = number; NTDB = National Trauma Data Bank; OR = odds ratio; PUF = Participant Use File; UTI = urinary tract infection.

Supplemental Table S6. E-Values for Key Outcomes (reference)

Outcome	Prevalence	OR	95% CI	E-value (Point)	E-value (CI)
Pulmonary Embolism (Primary)	0.0167	0.97	0.7–1.35	1.2	2.01
Deep Vein Thrombosis	0.0429	1.18	0.96–1.43	1.61	1.22
Venous Thromboembolism	0.0536	1.15	0.96–1.37	1.53	1.25
In-Hospital Mortality	0.0538	1.54	1.3–1.82	2.35	1.87

E-values quantify the minimum strength of association an unmeasured confounder would need with both the exposure and outcome to explain the observed association. Larger E-values indicate greater robustness. CI = confidence interval; OR = odds ratio; PE = pulmonary embolism.

Supplemental Table S7. Prespecified but Unexecuted Analyses (Methodological Note)

Prespecified analysis	Status	Reason not executed
Inclusion of inter-facility transfers	Prespecified; not executed	Transfers were excluded at cohort construction because transfer resets the recorded time from arrival to procedure, making time-to-decompression non-comparable with direct admissions.
Restriction to Level I trauma centers	Prespecified; not executed	Facility trauma-verification level is not available in the NTDB Participant Use File, so a Level I–only restriction cannot be operationalized from the registry.
All-S14 spinal cord injury codes	Prespecified; not executed	Requires source re-extraction under a broader SCI case definition (beyond S14.0xx/S14.1xx); not derivable from the locked analytic cohort and deferred to a future re-query.

These three analyses were prespecified in the analysis plan but were not executable from the registry as constructed; they are reported here rather than in the main robustness table (Table 5) so that the main table contains only estimable analyses. The previously prespecified “coded-decompression-only” sensitivity analysis is now subsumed by the primary cohort, which is restricted to coded cervical decompression by design and therefore no longer requires a separate sensitivity estimate. NTDB = National Trauma Data Bank; PUF = Participant Use File; SCI = spinal cord injury.

SUPPLEMENTAL FIGURES

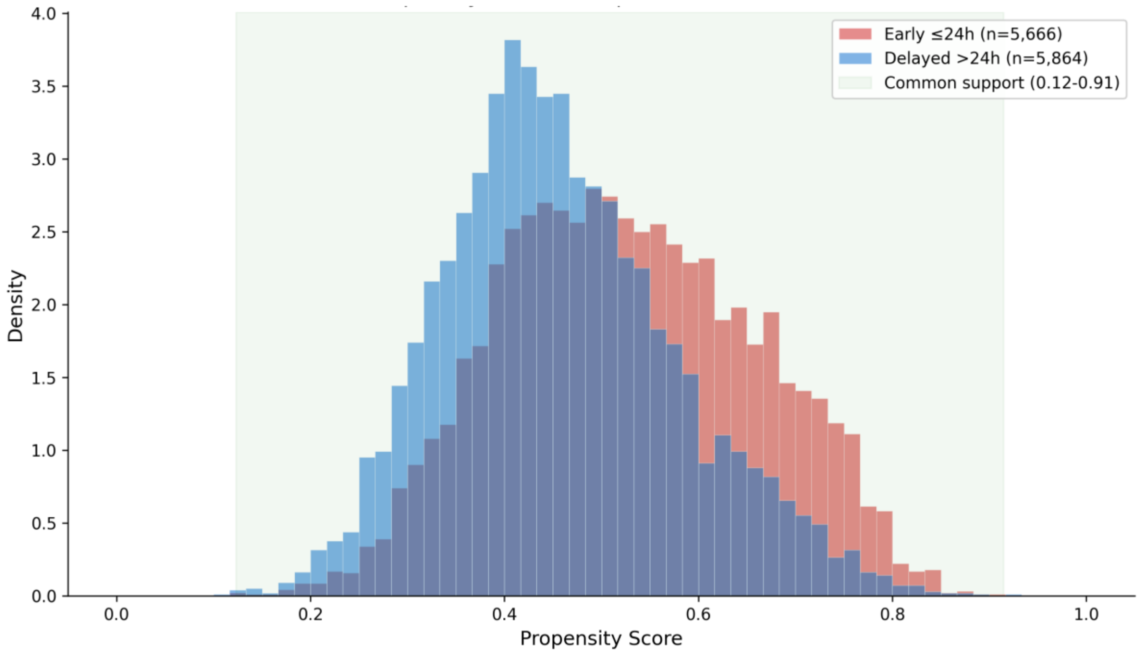

Supplemental Figure S1. Propensity Score Distribution Overlap. Density histograms of propensity scores for the early (red) and delayed (blue) decompression groups. The shaded region denotes the common support range used for matching, demonstrating adequate overlap between the two exposure groups.

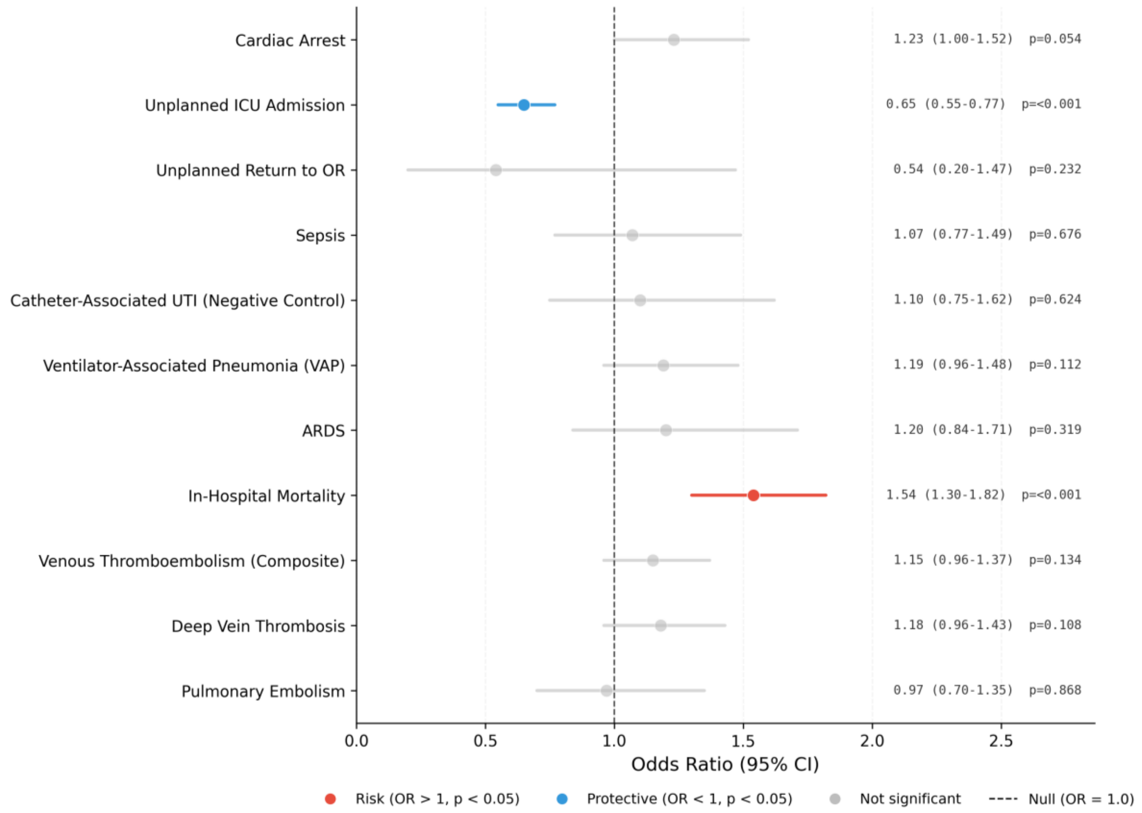

Supplemental Figure S2. Forest Plot of Secondary Outcomes. Odds ratios with 95% confidence intervals for secondary outcomes comparing early versus delayed decompression in the propensity score–matched cohort. Red markers denote statistically significant risk (OR > 1, $p < 0.05$), blue denote protective associations (OR < 1, $p < 0.05$), and gray denote non-significant comparisons. The dashed vertical line at OR = 1.0 indicates the null.
